# Biological Activities of Phenolic Compounds Present in Virgin Olive Oil

**DOI:** 10.3390/ijms11020458

**Published:** 2010-02-02

**Authors:** Sara Cicerale, Lisa Lucas, Russell Keast

**Affiliations:** School of Exercise and Nutrition Sciences, Deakin University, Melbourne, Australia; E-Mails: cicerale@deakin.edu.au (S.C.); ljlu@deakin.edu.au (L.L.)

**Keywords:** virgin olive oil, olive oil phenolic compounds, health benefits

## Abstract

The Mediterranean diet is associated with a lower incidence of atherosclerosis, cardiovascular disease, neurodegenerative diseases and certain types of cancer. The apparent health benefits have been partially ascribed to the dietary consumption of virgin olive oil by Mediterranean populations. Much research has focused on the biologically active phenolic compounds naturally present in virgin olive oils to aid in explaining reduced mortality and morbidity experienced by people consuming a traditional Mediterranean diet. Studies (human, animal, *in vivo* and *in vitro*) have demonstrated that olive oil phenolic compounds have positive effects on certain physiological parameters, such as plasma lipoproteins, oxidative damage, inflammatory markers, platelet and cellular function, antimicrobial activity and bone health. This paper summarizes current knowledge on the bioavailability and biological activities of olive oil phenolic compounds.

## 1. Introduction

A lower prevalence of non-communicable diseases such as cardiovascular disease and certain types of cancers have been demonstrated in countries residing in the Mediterranean region in comparison to other parts of the world [1–9]. This lowered incidence has been partially attributed to the regular intake of virgin olive oil as part of a traditional Mediterranean diet [3,5,9,10–17]. Dietary consumption of virgin olive oil in a Mediterranean diet typically ranges between 25–50 mL per day [18].

Virgin olive oil is produced from the first and second pressings of the olive fruit by the cold-pressing method (where no chemicals and only a small amount of heat are applied) and is composed of a glycerol fraction (making up 90–99% of the olive fruit) and a non-glycerol or unsaponifiable fraction (making up 0.4–5% of the olive fruit) which contains phenolic compounds [19]. Historically, the beneficial health effects of virgin olive oil intake were attributed to the glycerol fraction with its high concentration of monounsaturated fatty acids (MUFAs) (particularly oleic acid) [19]. However, several seed oils (including sunflower, soybean, and rapeseed) containing high quantities of MUFAs are ineffective in beneficially altering chronic disease risk factors [20,21]. Therefore, a substantial number of investigations examining the biological actions of olive oil phenolic compounds in the unsaponifiable fraction have been conducted. In this paper, the term virgin olive oil will be used to describe both extra virgin and virgin olive oil.

Studies conducted thus far (including human, animal, *in vivo* and *in vitro*) have demonstrated that olive oil phenolic compounds have positive effects on various physiological biomarkers, implicating phenolic compounds as partially responsible for health benefits associated with the Mediterranean diet [3,5,22–27]. Furthermore, olive oil phenolic compounds have been shown to be highly bioavailable, reinforcing their potential health promoting properties [28–33].

The phenolic fraction of virgin olive oil is heterogeneous, with at least 36 structurally distinct phenolic compounds identified. Variation in the phenolic concentration exists between differing virgin olive oils due to numerous factors including: variety of the olive fruit [34–42], region in which the olive fruit is grown [37], agricultural techniques used to cultivate the olive fruit [34,43,44], maturity of the olive fruit at harvest [35,39,44–48], and olive oil extraction, processing, storage methods and time since harvest [38,45,49–57]. Cooking methods have also been shown to alter phenolic concentrations in virgin olive oil [58,59,60]. Finally, research has shown that the analytical method used to quantify the concentration of phenolic compounds present in virgin olive oil has an influence on the reported concentration [61]. The factors mentioned above are not discussed in the current paper. For an extensive review on the matter, please see the paper by Cicerale and colleagues [62]. Therefore, the objective of the current paper was to review the bioavailability of olive oil phenolic compounds and the biological activities associated with them.

## 2. Bioavailability of Olive Oil Phenolic Compounds

The bioavailability of a compound refers to the degree in which it is extracted from a food matrix and absorbed by the body [63]. The majority of research regarding the bioavailability of olive oil phenolic compounds has focused on three major phenolics: hydroxytyrosol, tyrosol, and oleuropein, and in general, phenolics from virgin olive oil have been demonstrated to be readily bioavailable.

Research has shown that the phenolic compounds, hydroxytyrosol and tyrosol are absorbed after ingestion in a dose-dependent manner [30,64,65]. Tuck and colleagues [66] demonstrated increased bioavailability of hydroxytyrosol and tyrosol when administered as an olive oil solution compared to an aqueous solution. The differences in bioavailability have been suggested to be due to the high antioxidant content of virgin olive oil compared to water and this high antioxidant content may have protected the breakdown of phenolics in the gastrointestinal tract prior to absorption [66]. A further study found that absorption of administered ligstroside-aglycone, hydroxytyrosol, tyrosol, and oleuropein-aglycone was as high as 55–66% in humans [32]. Finally, oleuropein was demonstrated to be somewhat absorbed from isolated perfused rat intestine [67]. The mechanism by which absorption occurs with regards to olive oil phenolic compounds remains unclear. However, the different polarities of the various phenolics has been postulated to play a role in the absorption of these compounds [32]. For instance, the phenolics tyrosol and hydroxytyrosol are polar compounds and their absorption has been postulated to occur via passive diffusion [68]. The polar but larger phenolic, oleuropein-glycoside may be absorbed via a different mechanism to tyrosol and hydroxytyrosol. It has been proposed that oleuropein-glycoside may diffuse through the lipid bilayer of the epithelial cell membrane and be absorbed via a glucose transporter. Two additional mechanisms for oleuropein-glycoside absorption are potentially via the paracellular route or transcellular passive diffusion [67]. The phenolics, oleuropein and ligstroside-aglycones are less polar and currently there is no data available on their mechanism of absorption. Further research is required to substantiate the mechanisms of absorption for these phenolics and further investigate the mechanisms for other phenolic compounds.

Studies examining the quantity of phenolics excreted have also been carried out. A low quantity of phenolics present in urine after ingestion would indicate that these phenolics are readily absorbed. Excreted phenolics (mainly in the form of hydroxytyrosol and tyrosol) were determined to be 5–16% of the total ingested [32]. Excretion of approximately 24% of administered tyrosol was demonstrated in a study by Miro-Cases and colleagues [33]. Finally, Visioli and colleagues [30] reported the excretion of administered hydroxytyrosol and tyrosol to be between 30–60% and 20–22% of the total ingested by human subjects, respectively. The above findings demonstrate that humans absorb a significant portion (~40–95%, using hydroxytyrosol and tyrosol as proxy) of the dietary olive oil phenolic compounds they consume [32]. As most of the data is only based on three phenolics, more research is required on the excretion of other key phenolics in virgin olive oil.

The metabolism of olive oil phenolic compounds is important in determining their availability. If phenolics are broken down and converted to other phenolics this may have a notable effect on their bioavailability. Phenolic compounds, oleuropein-glycoside and oleuropein and ligstroside-aglycones are converted to hydroxytyrosol or tyrosol and excreted in urine [32]. Hydroxytyrosol and tyrosol themselves are sometimes conjugated to glucuronic acid and excreted in urine as glucuronides [30,32,65,69]. However, further work is needed in this area.

## 3. Olive Oil Phenolic Compounds and Health

**Figure 1.**
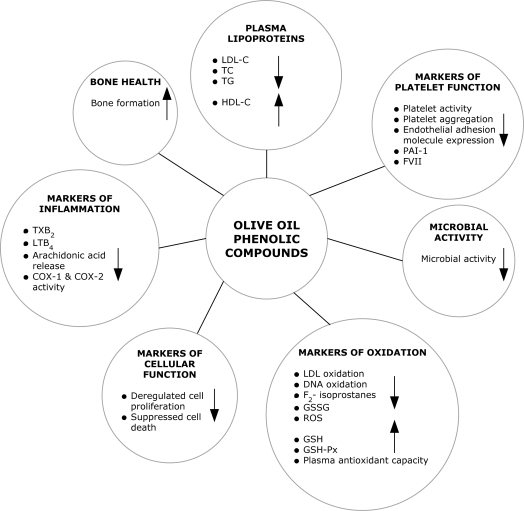
Biological activities of olive oil phenolic compounds (adapted from Cicerale *et al.* [62]).

Human and animal research have shown that olive oil phenolic compound’s possess important biological activities that may exert a preventative effect in regards to the development of chronic degenerative diseases. Figure 1 demonstrates the biological activities exerted by olive oil phenolic compounds. Table 1 briefly summarizes the findings of several human studies that have investigated the biological activities of olive oil phenolic compounds.

## 4. Olive Oil Phenolic Compounds and Their Beneficial Effect on Plasma Lipoproteins

Elevated levels of total cholesterol (TC) and low density lipoprotein cholesterol (LDL-C) have been established as risk factors for atherosclerosis, which is the primary cause of cardiovascular disease (CVD). However, on the other hand, elevated high density lipoprotein cholesterol (HDL-C) levels are believed to have protective, anti-inflammatory properties [85,86]. Data from a controlled human study containing 200 healthy male subjects, found a decrease in TC to HDL-C ratio with increasing phenolic content of the virgin olive oil consumed. An increase in HDL-C was also noted with increasing phenolic concentration of the oil [25]. Consumption of phenol rich virgin olive oils have resulted in increases in circulating HDL-C ranging between 5.1–6.7% in two further human studies [79,80]. Additionally, an earlier study showed a significant decrease in LDL-C after one week of phenol rich virgin olive oil consumption [87]. There are three human studies that showed olive oil phenolic compounds had no effect on blood lipid composition [77,82,84]. These studies used people with mild dyslipidemia and peripheral vascular disease (as opposed to healthy subjects used in the other studies) in two of the three studies and in the third study the authors propose the three week study period may not have been long enough to observe a treatment effect. However, it should be noted that in one study, four days was ample time to see a change in HDL-C [80].

Studies involving animals have demonstrated that ingestion of phenol rich virgin olive oil led to improvements in blood lipid profile. A study involving rabbits demonstrated a reduction in circulating TC and an increase in HDL-C upon virgin olive oil consumption. Furthermore, studies in rats have found that the intake of phenol rich virgin olive oil decreases TC, LDL-C and triglyceride (TG) levels [88] and substantially increases HDL-C concentrations [89].

## 5. Olive Oil Phenolic Compounds and Their Beneficial Effect on Lipid Oxidation

LDL oxidation (oxLDL) is considered to be a major risk factor for the development of atherosclerosis and CVD [90]. Oxidation of LDL causes damage to the vascular wall, stimulating macrophage uptake and formation of foam cells, which in turn result in the formation of plaque within the arterial wall [78,82,91,92]. Both human and animal *in vivo* studies have shown that the level at which LDL oxidizes, decreases linearly with increasing phenolic concentration [25,26,75,78–80,87,93,94]. Two further mechanistic studies, demonstrated that phenolic compounds are able to bind to LDL and the authors suggest that this may account for the increase in LDL resistance to oxidation [72,95]. Although it is important to note that there have been conflicting results from three short term studies suggesting that the phenolic content of the virgin olive oil does not play a role in the lowering of oxLDL [81,82,83]. In addition, *in vitro* studies have found that the phenolic compounds extracted from virgin olive oil inhibit the oxidation of LDL-C [40,96–98].

## 6. Olive Oil Phenolic Compounds and Their Beneficial Effect on Oxidative DNA Damage

Oxidative damage to DNA is a precursor for human carcinogenesis [99] and it is well known that oxygen radicals continually attack human cells [74]. Unless damage to these cells is counteracted, DNA damage may result, and such damage can lead to cancer development [74]. A randomized crossover intervention trial has shown that intake of phenol rich virgin olive oil decreases oxidative DNA damage by up to 30% compared to a low phenol virgin olive oil [74]. An additional study also demonstrated that after consumption of phenol rich virgin olive oil there was decreased urinary excretion of 8-oxo-deoxygyuanosine (8oxodG) a systemic marker of DNA oxidation [99,100].

In support of these findings, animal studies have also reported that a diet enriched with olive oil phenolic compounds has a protective effect against DNA damage [101,102]. In agreement with these findings, a recent *in vitro* study reported that olive oil phenolic compounds showed DNA oxidation preventative activity [103].

## 7. Olive Oil Phenolic Compounds and Their Beneficial Effect on Additional Markers of Oxidation

Oxidative stress produced by reactive oxygen species (ROS) has been linked to a number of diseases such as atherosclerosis, certain cancers and neurodegenerative diseases [90,104] and is considered as a by-product of aerobic metabolism. Goya *et al*. [105] treated a sample of human hepatoma HepG2 cells with hydroxytyrosol and found that there was a decrease in ROS production. Similarly olive oil phenolic compounds have been shown to scavenge ROS under natural and chemically simulated oxidative stress conditions [106–108]. A further study by Moreno *et al*. [109] demonstrated that tyrosol modulated ROS production, in murine macrophages [109].

Total plasma antioxidant activity has also been reported to increase in humans after the ingestion of olive oil phenolic compounds [73,74,77]. However, one study did not report an increase in the total blood antioxidant capacity [81].

Oxidative stress can be indicated by the presence of markers such as F_2_-isoprostanes, lipid peroxides (LPO), oxidized gluthathione (GSSG), reduced gluthathione (GSH) and glutathione peroxidase (GSH-Px). F_2_-isoprostanes are a result of the free radical induced peroxidation of arachidonic acid. LPO is more than likely a by-product of the oxidation of fatty acids [76] and depletion of the protective GSH precedes lipid oxidation and atherogenesis *in vivo* [110].

Human studies have shown beneficial effects of olive oil phenolic compounds on these aforementioned markers of oxidative stress. A randomized cross over study found the intake of a olive oil phenolic-enriched breakfast significantly lowered F_2_-isoprostane levels compared to a low phenolic-enriched breakfast [76]. Visioli and colleagues [29] demonstrated that consumption of a phenolic-rich virgin olive oil was associated with a significant decrease in urinary excretion of F_2_-isoprostanes. Another human study did not find an effect of olive oil phenolics on F_2_-isoprostane levels, this may be possibly due to the considerably low phenolic content of the oil administered (166 mg/kg) [77] compared to a phenolic content up 1950 mg/kg in the Visioli *et al.* study [29]. An animal study also found that administration of hydroxytyrosol-containing olive oil waste water to rats exposed to cigarette smoke, lowered F_2_-isoprostane levels significantly (*p* < 0.05) [64].

Covas and colleagues [25] found that phenol-rich virgin olive oil beneficially modulated the balance between GSH and GSSG, while Weinbrenner and colleagues [80] found an increase in GSH-Px after phenol-rich virgin olive oil administration in human subjects. Moreover, a decrease in LPO after olive oil phenolic administration has been noted. More recently, olive oil phenolic compounds sourced from olive mill waste water were found to increase GSH concentrations in human blood [111]. Two further studies have also shown that olive oil phenolic compounds reduce oxidative damage to both red blood cells and renal cells [108,112].

## 8. Olive Oil Phenolic Compounds and Their Beneficial Effect on Markers of Inflammation

Elevated concentrations of inflammation markers in serum are associated with increased cardiovascular risk [113]. Plasma thromboxane B_2_ (TXB_2_) and leukotriene B_4_ (LTB_4_) are known pro-inflammatory agents. TXB_2_ has the ability to increase blood platelet aggregation and LTB_4_ has a chemostactic effect on neutrophils, directing the cells to damaged tissue [73,114]. These inflammatory agents are known to produce the pain, redness and swelling associated with inflammation [115]. Bogani and colleagues [73] found a decrease in TXB_2_ and LTB_4_ concentrations with increasing phenolic concentration of the olive oil. These results were also in accordance with previous investigations [77,80,116].

The inflammatory markers, Interleukin-6 (IL-6) and C-reactive protein (CRP) have been shown to be predictors for CVD also [70]. IL-6 is a pro-inflammatory agent that stimulates inflammation in response to trauma and CRP generally rises when inflammation is present [70]. Fito and colleagues [70] found that consumption of olive oil phenolic compounds from a daily dose of virgin olive oil decreased the circulating concentrations of both IL-6 and CRP in 28 stable coronary heart patients.

*In vitro* study findings also support the anti-inflammatory capacity of olive oil phenolic compounds. Olive oil phenolics have been found to decrease arachidonic acid release and arachidonic acid metabolite synthesis *in vitro* and both of these are involved in the inflammatory process [109]. The olive oil phenolic compound, oleocanthal was shown to inhibit cyclooxygenase-1 (COX-1) and cyclooxygenase-2 (COX-2) activity (both involved in the inflammatory process) in the same way as the anti-inflammatory drug, ibuprofen does [117]. Inhibition of COX enzymes results in the reduction of arachidonate to the eicosanoids, prostaglandins and thromboxane, in the inflammatory pathway [114,118].

## 9. Olive Oil Phenolic Compounds and Their Beneficial Effect on Platelet Function

Blood platelets have been demonstrated to play a role in CVD and atherosclerosis development. Continual damage to the vascular epithelium results in the development of lesions and these lesions stimulate endothelial adhesion molecule expression, platelet activity and aggregation [119,120]. Circulating monocytes are attracted by these particular molecules and adhere to the endothelium and differentiate into macrophages, which in turn scavenge LDL and triglyceride (TG) rich lipoproteins, becoming foam cells and forming fatty streaks [121].

Olive oil phenolic compounds have been shown to inhibit endothelial adhesion molecule expression upon incubation with human umbilical vein endothelial cells [27]. They also have been demonstrated to inhibit human platelet activity *in vitro* [122]. The phenolic compound, hydroxytyrosol has been noted to completely inhibit platelet aggregation in human blood (*in vitro*) in the range of 100–400 μM [123]. A more recent investigation, demonstrated that a number of olive oil phenolic compounds (such as oleuropein aglycone and luteolin) also were potent inhibitors of platelet aggregation [124]. Virgin olive oil containing a high content (400 mg/kg) of phenolic compounds has been demonstrated to also decrease plasminogen activator inhibitor-1 (PAI-1) and factor VII (FVII). Both PAI-1 and FVII are pro-coagulant factors that have been linked to the development of coronary heart disease (CHD) [71]. Furthermore, olive oil phenolics have been shown to decrease homocysteine, which has been linked to increased adhesiveness of the endothelium [125].

## 10. Olive Oil Phenolic Compounds and Their Beneficial Effect on Cellular Function

Cell proliferation and suppressed cell death are underlying factors for tumor formation and progression [126]. Research to date has shown that the olive oil phenolic, hydroxytyrosol inhibits cell proliferation in human promyelocytic HL60 leukemia cells and in human colon cancer lines [127–129]. Hashim and colleagues [130] demonstrated a dose-related inhibition of colon cancer cell invasion by olive oil phenolic compounds. Also, hydroxytyrosol has been found to exert strong anti-proliferative effects against human colon adenocarcinoma cells [131]. More recently, research using MCF-7 and SKBR3 breast cancer cells showed that phenolic compounds inhibit cell growth in these cell lines in a dose dependent manner and reduce expression of the HER2 oncogene which plays a integral role in malignant transformation, tumorigenesis, and metastasis [132–134]. Furthermore, oleuropein and hydroxytyrosol have been show to induce cell death of MCF-7 human breast cancer cells [135]. Olive oil phenolics have also been found to improve cell integrity and viability in CaCo2 cells [128,136].

The olive oil phenolic compound, oleocanthal has been implicated in the reduced incidence of Alzheimer’s disease in Mediterranean populations via two mechanisms. First, in Alzheimer’s disease a microtubule-associated protein (Tau) involved in the promotion of microtubule assembly and stability begin to aggregate into neurofibrillary tangles. Li and colleagues [137] demonstrated that oleocanthal inhibits tau aggregation. Second, beta-amyloid (Aβ) oligomers (also referred to as ADDLs) have also been suggested to be involved in the development of Alzheimer’s disease. These ADDLs are believed to bind to postsynaptic sites and cause synaptic and neuronal loss. Pitt and colleagues [138] have demonstrated that oleocanthal has the capacity to alter the oligomerization state of ADDLs whilst protecting neurons from the synaptopathological effects of ADDLs. Thus, oleocanthal protects neurons from ADDL induced synaptic deterioration and additionally promotes the antibody clearance of ADDLs [138]. A further study demonstrated the neuro-protection of hydroxytyrosol in rat brains. In this study it was found that hydroxytyrosol reduced lactate dehydrogenase activity which has been closely related with a reduction in brain lipid peroxidation [139].

## 11. Olive Oil Phenolic Compounds and Their Beneficial Effect on Microbial Activity

*In vitro* research has shown that olive oil phenolic compounds have antimicrobial properties. Particularly, the phenolic compounds, oleuropein, hydroxytyrosol and tyrosol have demonstrated potent antimicrobial activity against several strains of bacteria responsible for intestinal and respiratory infections [140]. Romero and colleagues [141] found that the dialdehydic form of decarboxymethyl ligstroside is not hydrolyzed in the stomach and therefore aids in inhibiting the growth of *Helicobacter pylori* bacteria. *Helicobacter pylori* bacteria are linked to the development of peptic ulcers and some types of gastric cancer. Hydroxytyrosol and oleuropein have also been shown to be cytotoxic to a large number of bacterial strains [142].

## 12. Olive Oil Phenolic Compounds and Their Beneficial Effect on Bone

One study to date has investigated the effect of olive oil phenolic compounds on bone [143]. In this study, both tyrosol and hydroxytyrosol increased bone formation in rats significantly. Further studies are now required to substantiate these findings.

## 13. Conclusions

In conclusion, olive oil phenolic compounds are highly bioavailable in humans. The high bioavailability of such compounds lends support to evidence that these phenolic components exert beneficial effects on health. Although, the beneficial health effects of virgin olive oil ingestion are well known, it is only recent that that the biological properties of olive oil phenolic compounds have been investigated. In experimental studies (*in vivo* and *in vitro*), olive oil phenolic compounds have been shown to beneficially alter lipid composition, platelet and cellular function, microbial activity and bone formation, as well as reduce oxidative damage and inflammation. The modes of action detailed in the paper, may explain the low rate of diet-related diseases amongst populations residing in the Mediterranean region. For example, the anti-atherogenic effects associated with the ingestion of virgin olive oil may explain the low rate of cardiovascular disease in Mediterranean populations. Since DNA oxidative damage is a mechanism underlying cancer development, the protective effects of olive oil phenolic compounds may explain some of the differences in cancer incidence between Mediterranean populations and other populations in the world. The anti-inflammatory effects that arise from the ingestion of olive oil phenolic compounds have been shown to provide protection against diseases marked by an inflammatory component. This may, along with other modes of action, partly explain the low rate of CVD mortality and certain types of cancer in populations residing in the Mediterranean. Olive oil phenolic compounds may also be useful in the treatment of some infectious diseases. Finally, although more studies are required, findings demonstrating the beneficial effects of olive oil phenolics in relation to bone health, may aid in partly explaining the low incidence of osteoporosis in populations residing in the Mediterranean area. These biological properties may have a significant impact on population health through the reduction in incidence of chronic degenerative disease development.

## Figures and Tables

**Table 1.** Randomized, crossover, controlled, human studies on the effect of olive oil phenolic compounds on biomarkers of health (adapted from Cicerale *et al.* [62]).

**Treatment**	**Subject number**	**Olive oil phenolic concentration**	**Study design**	**Investigated biomarker**	**Key findings**	**Ref.**
High phenolic concentration *vs.* low phenolic concentration olive oil	28 coronary heart disease subjects	161 *vs.* 14.67 mg/kg	3 week, crossover	IL-6, C-reactive protein, sICAM-1, sVCAM-1 and plasma lipids	Interleukin-6 and C-reactive protein decreased after phenol-rich olive oil consumption. However, no changes in soluble intercellular (sICAM-1) and vascular adhesion (sVCAM-1) molecules and lipid profile were observed.	[70]
High phenolic concentration *vs.* low phenolic concentration-enriched breakfast	21 hypercholesterolemic subjects	400 *vs.* 80 mg/kg	Acute dose, crossover	FVIIa and PAI-1	Concentrations of FVIIa increased less and PAI-1 activity decreased more after the high phenolic breakfast than after the low phenolic breakfast.	[71]
High phenolic concentration *vs.* moderate phenolic concentration *vs.* poor phenolic concentration olive oil	30 healthy subjects	825 *vs.* 370 *vs.* 0 μmol CAE/kg	3 week, crossover	Plasma lipids and oxLDL	An increase in phenolic content of LDL-C and decrease in oxLDL was noted after consumption of oil rich in phenolic compounds.	[72]
High phenolic concentration *vs.* low phenolic concentration olive oil *vs.* corn oil	12 healthy subjects	607 *vs.* 16 *vs.* 0 mg/kg	Acute dose, crossover	Plasma TXB_2_, plasma LTB_4_ and plasma antioxidant capacity	Decrease in TXB_2_ and LTB_4_ with increasing phenolic content of olive oil and concomitant increase in plasma antioxidant capacity with increased phenolic content of olive oil.	[73]
High phenolic concentration *vs.* low phenolic concentration olive oil	10 healthy subjects	592 *vs.* 147 mg/kg	8 week, crossover	Oxidative DNA damage and plasma antioxidant capacity	A reduction in DNA damage with the consumption of a phenol-rich olive oil diet was demonstrated. No difference was seen in plasma antioxidant capacity.	[74]
High phenolic concentration *vs.* moderate phenolic concentration *vs.* low phenolic concentration olive oil	200 healthy subjects	366 *vs.* 164 *vs.* 2.7 mg/kg	3 week, crossover	Plasma lipids, plasma oxLDL, plasma F2-isoprostanes, GSH and GSSG	A linear increase in HDL-C was observed for low-, medium-, and high phenolic olive oil. Furthermore, TC to HDL-C ratio decreased linearly with the increasing phenolic content of the olive oil. OxLDL decreased linearly with increasing phenolic content of the olive oil and TG levels decreased for all olive oils. Oxidative stress markers indicated by GSH and GSSG decreased linearly with increasing phenolic content.	[25]
High phenolic concentration *vs.* moderate phenolic concentration *vs.* low phenolic concentration olive oil	12 healthy subjects	366 *vs.* 164 *vs.* 2.7 mg/kg	Acute dose, crossover	Plasma F2-isoprostanes and plasma oxLDL	All olive oils promoted postprandial oxidative stress indicated by increased F2-isoprostanes, however, the degree of LDL oxidation decreased as the phenolic content in administered oil increased.	[75]
High phenolic concentration *vs.* low phenolic-enriched breakfast	21 hypercholesterolemic subjects	400 *vs.* 80 mg/kg	Acute dose, crossover	Plasma LPO and plasma F2-isoprostanes	Decrease in LPO and F2-isoprostanes with intake of the phenol-enriched breakfast compared to low phenol-enriched breakfast.	[76]
High phenolic concentration *vs.* low phenolic concentration olive oil	22 mild dyslipidemic subjects	166 *vs.* 2 mg/kg	49 day, crossover	Plasma lipids, plasma TXB2, plasma antioxidant capacity and urinary F2-isoprostanes	Plasma TXB2 decreased with phenol-rich olive oil supplementation. Plasma antioxidant capacity increased after phenol-rich olive oil administration. No effect on urinary F2-isoprostanes and plasma lipids between phenol-rich and phenol-poor olive oil were observed.	[77]
High phenolic concentration *vs.* low phenolic concentration olive oil	43 coronary heart disease subjects	161 *vs.* 14.67 mg/kg	3 week, crossover	Plasma oxLDL, plasma LPO and whole blood GSH-Px	Decrease in oxLDL and LPO and increase in GSH-Px upon phenol-rich olive oil consumption.	[78]
High phenolic concentration *vs.* moderate phenolic concentration *vs.* low phenolic concentration olive oil	30 healthy subjects	150 *vs.* 68 *vs.* 0 mg/kg	3 week, crossover	Plasma lipids and oxLDL	Sustained consumption of phenol-rich olive oil was more effective in protecting LDL from oxidation and in raising HDL-C than olive oils with lesser quantities of phenolics.	[79]
High phenolic concentration *vs.* moderate phenolic concentration *vs.* low phenolic concentration olive oil	12 healthy subjects	486 *vs.* 133 *vs.* 10 mg/kg	4 day, crossover	Plasma lipids, plasma oxLDL, plasma GSH-Px and urinary 8-oxo-dG	Short-term consumption of phenol-rich olive oil decreased plasma oxLDL, urinary 8-oxo-dG, and increased plasma HDL-C and GSH-Px, in a dose-dependent manner with the increasing phenolic content of the olive oil administered.	[80]
High phenolic concentration *vs.* low phenolic concentration olive oil	25 healthy subjects	21.6 *vs.* 3.0 mg/kg	3 week, crossover	Plasma antioxidant capacity and oxLDL	Plasma antioxidant capacity and oxLDL did not differ significantly between the phenol-rich and phenol-poor olive oil.	[81]
High phenolic concentration *vs.* low phenolic concentration olive oil	46 health subjects	308 *vs.* 43 mg/kg	3 week, crossover	Plasma lipids, plasma oxLDL and plasma LPO	No effect on plasma lipids, oxLDL, and LPO were noted between the phenol-rich and phenol-poor olive oils.	[82]
Olive oil with different phenolic concentrations	6 healthy subjects	1950 *vs.* 1462.5 *vs.* 975 *vs.* 487.5 mg/kg	Acute dose, cross over	Urinary F2-isoprostanes	A dose-dependent decrease in urinary excretion of F2-isoprostanes was noted upon administration of phenol-rich olive oil.	[29]
High phenolic concentration *vs.* low phenolic concentration olive oil	14 healthy subjects	303 *vs.* 0.3 mg/kg	4 week, crossover	Plasma oxLDL and serum antioxidant capacity	Increase in plasma antioxidant capacity but no change in oxLDL.	[83]
High phenolic concentration *vs.* low phenolic concentration olive oil	24 peripheral vascular disease subjects	800 *vs.* 60 mg/kg	12 week, crossover	Plasma lipids and plasma oxLDL	A lower oxLDL was noted in subjects after administration of phenol-rich olive oil. No difference in plasma lipids was observed.	[84]

## Abbreviations:

ADDL: beta-amyloid oligomers
CHD: coronary heart disease
COX-1: cyclooxygenase-1
COX-2: cyclooxygenase-2
CRP: C-reactive protein
CVD: cardiovascular disease
DNA: deoxyribonucleic acid
FVII: factor VII
g: gram
GSSG: glutathionedissulfide
GSH: reduced glutathione
GSH-Px: glutathione peroxidise
HDL-C: high density lipoprotein cholesterol
IL-6: interleukin-6
LDL: low density lipoprotein
LDL-C: low density lipoprotein cholesterol
LPO: lipid peroxidation
LTB_4_: leukotriene B_4_
MUFA: monounsaturated fatty acid
μM: micromolar
μg: microgram
oxLDL: low density lipoprotein oxidation
8-oxo-dg: 8-oxo-2’-deoxyguanosine
PAI-1: plasminogen activator inhibitor-1
ROS: reactive oxygen species
sICAM-1: soluble intercellular molecules
sVCAM-1: soluble vascular adhesion molecules
Tau: microtubule-associated protein
TC: total cholesterol
TG: triglyceride
TXB_2_: thromboxane B2
